# Kineplastic Amputation

**Published:** 1918-10

**Authors:** 


***Kineplastic Amputation.*** By Dr. VINCENT GAUDEANS of New York. Abs. from *Annals of Surgery*, April 1918.

The writer has gone into the literature carefully and furnishes a complete bibliography of the subject of kineplastic amputations. He thinks that Vanghetti's method is especially adapted to the upper limb and can be used in every case by forming " knobs " or " loops " at the end of the stump. These stumps are to be covered with healthy skin and form a basis for later adaptation of apparatus, or, as he calls them, " plastic motor ". The loop is made by the natural or artificial union of muscles and tendons forming a space through which a ring can be adapted to transmit contraction outside.

The knob is an enlargement of tendons or muscles endings, formed by the surgeons either twisting a number of tendons, or by using a bone eminence where the tendon is attached.

The after-care is extremely important in order to maintain proper functioning of muscles.

Secondary kineplastic operation is desirable and should be performed in institutions where the artificial limb may be fitted at the same time.

1. The kineplastic amputation (Vanghetti) is such a modification of the operative technic that it makes possible the transmission of voluntary movements to the prothesis, thus realizing a true vitalization of the amputated members.

2. This method should be applied whenever possible, especially in the upper limb.

3. In performing amputation of the upper limb, one should always have in mind the possibility of a future kineplastic transformation of the stump.

4. Secondary kineplastic operation should be favored and possibly performed in institutions where, at the same time, the artificial member could be constructed and fitted.

